# Extracellular vesicles: molecular messengers and new therapeutic targets in acute myocardial infarction

**DOI:** 10.3389/fimmu.2025.1598407

**Published:** 2025-06-26

**Authors:** Hao Wu, Jinyi Xue, Qiuyu Liu, Zhenxun Wan, Lingling Liang, Shihan Sun, Ping Liu, Mingtai Chen, Mengnan Liu

**Affiliations:** ^1^ Affiliated Traditional Chinese Medicine Hospital, Southwest Medical University, Luzhou, China; ^2^ School of Pharmacy, Southwest Medical University, Luzhou, China; ^3^ Shenzhen Traditional Chinese Medicine Hospital, Guangzhou University of Chinese Medicine, Shenzhen, China

**Keywords:** extracellular vesicles, acute myocardial infarction, therapeutic targets, cardiac repair, biomarkers, immune modulation

## Abstract

Extracellular vesicles (EVs) are nanoscale particles secreted by cells, encapsulating a variety of biomolecules, and have emerged as significant players in the pathophysiology of acute myocardial infarction (AMI). These vesicles exhibit both detrimental and therapeutic effects. On one hand, EVs contribute to AMI progression by promoting apoptosis, exacerbating inflammatory responses, and impairing angiogenesis. On the other hand, they facilitate cardiac repair by enhancing neovascularization, mitigating programmed cell death, and inhibiting fibrosis. This review provides a comprehensive overview of EV biogenesis, release mechanisms, and their dual regulatory roles in AMI, emphasizing the complex interplay of EVs in myocardial injury. Additionally, it explores the potential of EVs as diagnostic biomarkers and therapeutic delivery vehicles, highlighting their importance in advancing diagnostic and therapeutic strategies. By elucidating the multifaceted roles of EVs, this review aims to establish a foundation for their clinical translation, improve their applicability in precision medicine, and explore the promising potential in cardiovascular disease treatment.

## 1 Introduction

Acute myocardial infarction (AMI) remains one of the leading causes of high mortality and disability in cardiovascular diseases. Despite significant advancements in medical technologies, such as timely thrombolysis, percutaneous coronary intervention (PCI) for vascular reperfusion, and the standardized use of antithrombotic, antiplatelet, and prognostic-improving pharmacotherapies, AMI continues to be a significant cardiovascular disorder closely associated with global mortality (1). The continuing clinical need for innovative and effective treatment strategies highlights the urgency of exploring novel treatment options. Extracellular vesicles (EVs) are naturally secreted, non-replicative, nucleus-free particles encased in a lipid bilayer. Depending on their biogenesis, biophysical properties, and receptor composition, EVs can be classified into various subtypes, including exosomes, microvesicles, apoptotic bodies, exosome-like vesicles, migrasomes, and ectosomes, with exosomes, microvesicles, and apoptotic bodies being the most extensively studied (2). EVs encapsulate a broad array of biomolecules derived from their parent cells, such as proteins, mRNA, microRNA, lipids, and small-molecule metabolites. These biomolecules can be transferred to recipient cells, thereby mediating intercellular communication and regulation. Secreted by various cell types and tissues, EVs exhibit lower immunogenicity, reduced tumorigenic potential, and enhanced stability, making them promising candidates for therapeutic applications (3). Emerging research indicates that EVs play pivotal roles in regulating diverse physiological and pathological processes and act as key mediators of intercellular signaling, presenting a breakthrough avenue for disease treatment. Their potential application in AMI therapy has sparked increasing interest. This review provides an in-depth discussion of the therapeutic and biomarker potential of EVs derived from various cell types in AMI. By exploring their roles in promoting angiogenesis, alleviating myocardial fibrosis, improving cardiac function, modulating inflammation, and regulating immune responses, this review aims to offer insights into the mechanistic underpinnings of EVs in AMI and promote their clinical translation as a promising strategy for cardiovascular therapy.

## 2 Biogenesis, release, and uptake of extracellular vesicles

**Figure 1:**
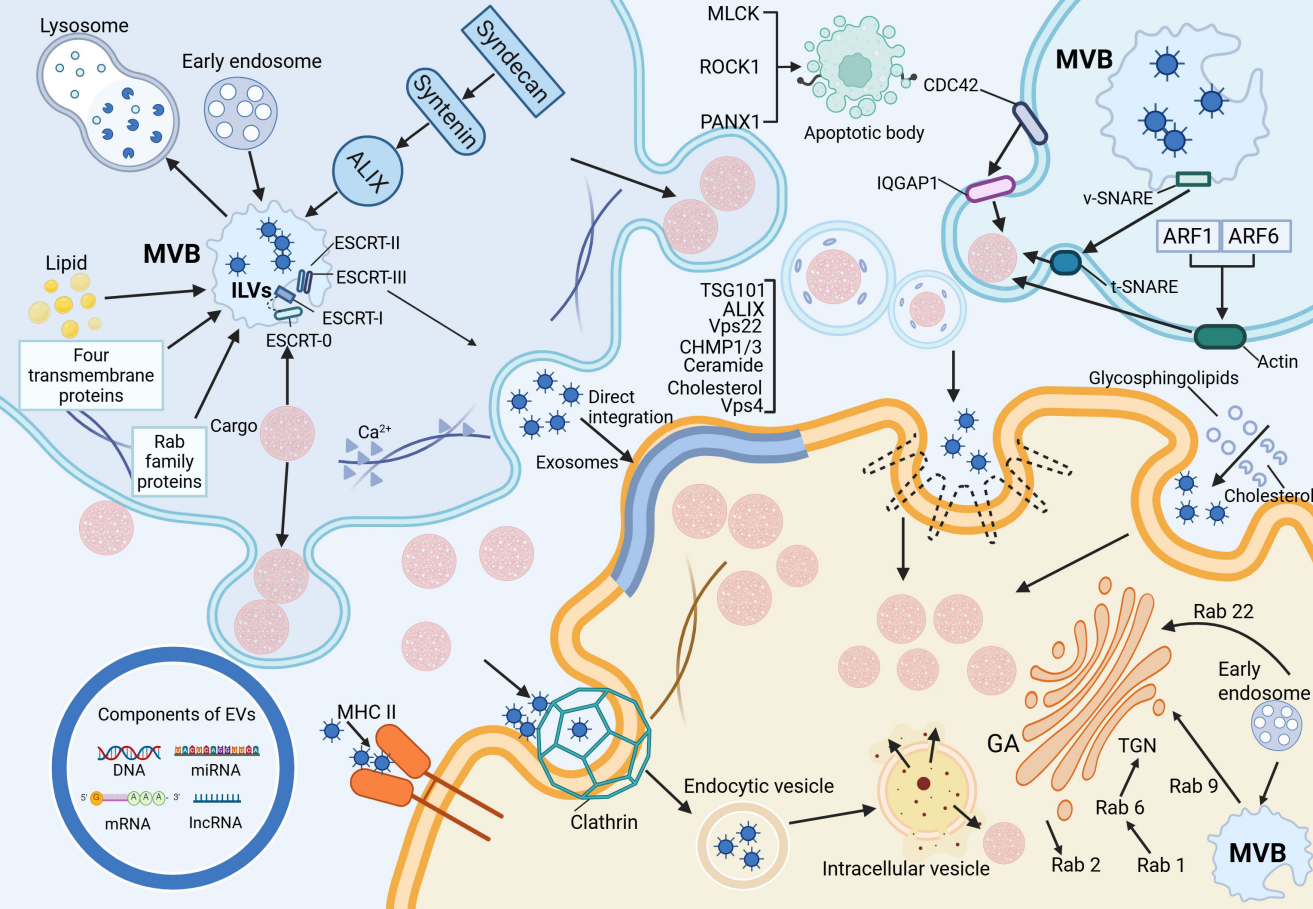
Biogenesis, release, and uptake of extracellular vesicles.

As illustrated in **Figure 1**, the left figure summarizes the biogenesis pathways of exosomes, microvesicles, and apoptotic bodies, as well as the regulatory mechanisms governing EV release, including cytoskeletal remodeling and membrane fusion events. The right figure illustrates the primary mechanisms by which recipient cells internalize EVs, such as membrane fusion, receptor–ligand interactions, and various endocytic pathways. By transporting bioactive molecules including proteins and nucleic acids, EVs play a pivotal role in mediating intercellular communication. The composition and functional properties of EVs can vary significantly, even when secreted by the same cell type under different environmental conditions. Moreover, different classes of EVs follow different biogenetic pathways, which further contributes to their heterogeneity and specific functions (4).

### 2.1 Biogenesis of extracellular vesicles and exosome formation

EVs are widely distributed across various biological fluids, including plasma, serum, saliva, amniotic fluid, breast milk, and urine, and are also secreted into cell culture media (5). Among the different EV subtypes, exosomes originate from intraluminal vesicles (ILVs) within multivesicular bodies (MVBs), which fuse with the plasma membrane to release ILVs into the extracellular space (6).

Exosome formation primarily occurs through the endosomal pathway. Early endosomes undergo inward budding of their limiting membrane, generating ILVs within MVBs. These MVBs may either fuse with lysosomes for degradation or the plasma membrane to release ILVs as exosomes (7). The biogenesis of ILVs is regulated by both endosomal sorting complex required for transport (ESCRT) -dependent and ESCRT-independent mechanisms. The ESCRT-dependent pathway involves four protein complexes (ESCRT-0, -I, -II, and -III) along with accessory proteins that coordinate membrane remodeling and vesicle scission (8). ESCRT-0 recognizes and binds ubiquitinated cargo, recruiting ESCRT-I, which subsequently engages ESCRT-II to drive membrane invagination and ILV formation. ESCRT-III facilitates vesicle scission, a process that ALG-2-interacting protein X (ALIX)-mediated recruitment can further regulate. The ESCRT-independent pathway relies on alternative mechanisms, including the Syndecan-Syntenin-ALIX axis, lipid rafts, tetraspanins, and Rab family GTPases. Together, these factors regulate ILV formation and cargo sorting, reflecting the complexity and diversity of exosome biogenesis (9).

### 2.2 Mechanisms underlying the formation of microvesicles and apoptotic bodies

The biogenesis of microvesicles (MVs) fundamentally differs from that of exosomes, as MVs are formed through the direct outward budding of the plasma membrane, with a size range of 100–1000 nm (10). Their generation shares mechanistic similarities with ILV formation, involving both ESCRT-dependent and lipid-dependent pathways. Notably, the suppression of ESCRT-associated proteins, such as ALIX, TSG101, Vps22, CHMP1/3 (charged multivesicular body protein 1/3), and Vps4, results in a marked reduction in MV secretion (11). In addition, lipid components such as ceramides and cholesterol play regulatory roles in MV formation. A key driver of MV biogenesis is Ca²^+^-dependent cytoskeletal remodeling, which facilitates membrane deformation and vesicle budding. In contrast, apoptotic bodies (ABs) are distinct from other EV subtypes, as they are generated exclusively during programmed cell death and serve as hallmarks of apoptosis (12). Their size, ranging from 50 to 5000 nm, distinguishes them from the continuous release of EVs by viable cells. ABs emerge during the disassembly of apoptotic cells, wherein nuclear and cytoplasmic fragments are rapidly enclosed within densely packed membrane-bound vesicles of varying sizes (13). Studies have identified the involvement of specific kinases in AB formation, including myosin light chain kinase (MLCK), Rho-associated kinase (ROCK1), and pannexin 1 (PANX1), a plasma membrane channel protein. These molecular regulators orchestrate cytoskeletal reorganization and membrane dynamics required for AB biogenesis, further highlighting their mechanistic divergence from other EV subtypes (14).

### 2.3 Release of extracellular vesicles

Exosome secretion occurs following the fusion of MVBs with the plasma membrane, a process that relies on the soluble N-ethylmaleimide-sensitive factor attachment protein receptor (SNARE) complex (15). During this event, v-SNARE proteins on the MVB membrane interact with t-SNARE proteins on the plasma membrane, forming a functional SNARE complex that facilitates membrane fusion and subsequent release of ILVs. Key molecular regulators of exosome release include VAMP7, a v-SNARE-associated protein associated with membrane transport and cell migration, which modulates EV secretion in specific cell types (16). Additionally, SNAP23, a t-SNARE protein, and YKT6, a member of the SNARE family, serve as essential mediators of exosomal release. In contrast, the shedding of MVs is governed by the Rho family of small GTPases and Rho-associated kinase (ROCK) signaling pathways. Among these, CDC42, a key Rho-family GTPase, acts as a central hub integrating multiple regulatory signals for MV biogenesis (17). Activation of CDC42 by GTP promotes MV release via its downstream effector, IQGAP1 (IQ-domain GTPase-activating protein 1), which facilitates membrane budding. Simultaneously, CDC42 sustains epidermal growth factor (EGF) signaling by inhibiting receptor endocytosis, further enhancing MV secretion. Additionally, ARF1 and ARF6, small GTP-binding proteins, contribute to MV release by activating RhoA, which drives actomyosin contraction and promotes vesicle shedding (18).

### 2.4 Cargo, uptake, and intercellular communication of extracellular vesicles

EVs are critical mediators of intercellular communication, carrying a wide range of biomolecular “cargo,” including proteins, lipids, and nucleic acids (such as DNA, mRNA, miRNA, and lncRNA) (19). The lipid bilayer encapsulating these molecules ensures their stability and integrity, allowing for the efficient transfer of information between cells (12). At the same time, this ability to reflect both physiological and pathological changes has led to the recognition of EVs as potential clinical diagnostic biomarkers. EVs interact with target cells through three primary mechanisms. First, exosomes and target cells directly interact via ligands and receptors (such as proteins, sugars, and lipids) on their respective membranes, initiating a cascade of signaling events (20). For instance, dendritic cells can transfer membrane proteins, like Major Histocompatibility Complex II (MHC II), to homologous T cells via exosomes, thereby playing a role in immune regulation (21). Second, the lipid bilayer of EVs can fuse directly with the target cell membrane, releasing their internal contents (such as proteins and RNA) into the cytoplasm, thus effectively transferring information. Third, EVs can be internalized by target cells through endocytosis, which includes clathrin-dependent endocytosis, caveolin-dependent endocytosis, macropinocytosis, phagocytosis, and lipid raft-mediated endocytosis (22). In clathrin-dependent endocytosis, clathrin assembles around membrane receptors to form a hexagonal and triangular lattice structure that encases the receptors and internalized substances, leading to the formation of clathrin-coated vesicles, which then fuse with intracellular vesicles to release their contents (23). Caveolin-mediated endocytosis, distinct from clathrin-mediated endocytosis, involves RhoA-dependent and Cdc42-mediated processes. These pathways are distinguished by their sensitivity to the biochemical properties of the cargo and the specificity of the involvement of adaptor proteins (11). External cholesterol and sphingolipids selectively stimulate caveolin-dependent endocytosis. Unlike the other two mechanisms, macropinocytosis and phagocytosis form larger vesicles (24). In macropinocytosis, the cell membrane undergoes folding to form large, irregular vesicles that engulf extracellular fluid and materials, whereas phagocytosis relies on receptor-ligand interactions to internalize particles. Lipid rafts, composed of cholesterol, sphingolipids, and receptor proteins, mediate endocytosis influenced by the lipid composition of these microdomains (25).

### 2.5 miRNA-mediated intercellular communication mechanisms: canonical and non-canonical pathways

Accumulating evidence indicates that exosome-associated miRNAs play crucial roles in various cardiac pathophysiological processes, particularly in myocardial repair and the regulation of fibrosis following ischemic injury, by modulating the function of recipient cells (26). Traditionally, miRNAs are thought to exert their effects via canonical mechanisms, primarily through complementary binding to the 3′ untranslated region (3′UTR) of target mRNAs, thereby repressing translation or promoting mRNA degradation, ultimately influencing downstream signaling pathways and cellular functions (27). However, an increasing number of studies have revealed that certain miRNAs can also mediate biological effects through non-canonical pathways. For instance, miR-21, miR-29a, and members of the let-7 family have been shown to act as endogenous ligands for Toll-like receptors 7 and 8 (TLR7/8), triggering inflammatory or stress responses in recipient cells (28). These findings suggest that exosomal miRNA-mediated intercellular communication extends beyond the regulation of gene expression and may also involve immune recognition, apoptosis, and metabolic regulation, thus unveiling a broader and more complex spectrum of biological effects.

## 3 The dual role of extracellular vesicles in acute myocardial infarction

**Figure 2:**
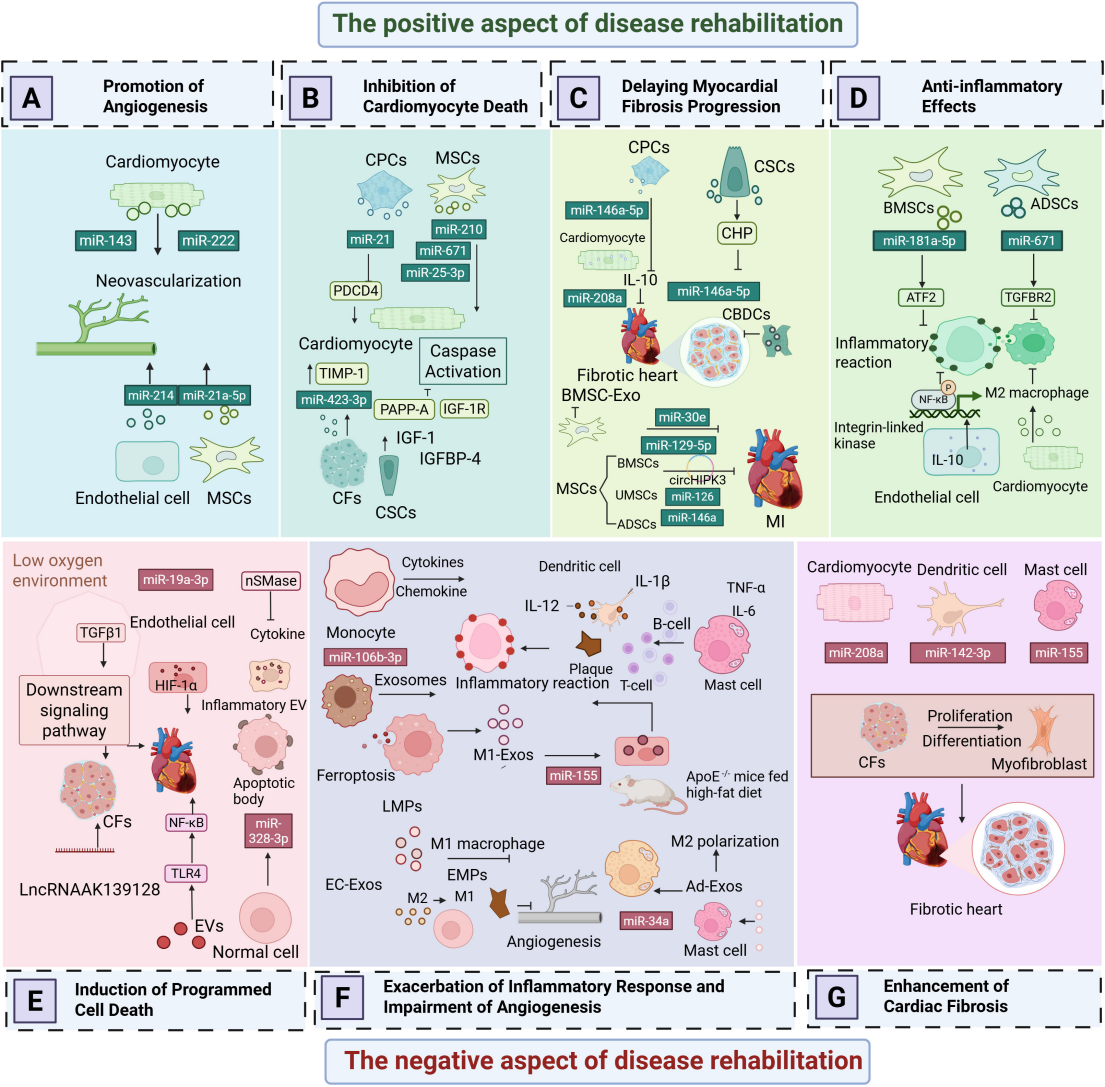
The dual role of extracellular vesicles in acute myocardial infarction. **(A)** Promotion of Angiogenesis. **(B)** Inhibition of Programmed Cardiomyocyte Death. **(C)** Delaying Myocardial Fibrosis Progression. **(D)** Anti-Inflammatory Effects. **(E)** Induction of Programmed Cell Death. **(F)** Exacerbation of Inflammatory Responses and Impairment of Angiogenesis. **(G)** Enhancement of Cardiac Fibrosis.

AMI is most commonly caused by intraluminal occlusion of the coronary artery due to atherosclerosis and the rupture and erosion of unstable plaques (29). When the blood supply is persistently reduced or completely interrupted, a large portion of the myocardium undergoes coagulative necrosis, accompanied by congestion, edema, and extensive infiltration of inflammatory cells in the myocardial interstitium (30). These pathological changes lead to a significant decline in myocardial contractility and a sudden reduction in cardiac output. Consequently, controlling excessive inflammatory responses, inhibiting myocardial apoptosis and necrosis, preventing ventricular fibrosis, and promoting vascular regeneration have emerged as potential therapeutic strategies to improve the prognosis of AMI patients. To gain a more comprehensive understanding of the negative effects of EVs in AMI, (**Figures 2E–G**) and **Table 1** illustrate various detrimental impacts of EVs associated with myocardial injury, including their roles in promoting programmed cell death, exacerbating inflammatory responses, and enhancing cardiac fibrosis. In contrast, (**Figures 2A–D**) and **Table 2** reveal the mechanisms through which EVs improve the prognosis of AMI. Exploring how to modulate the function of EVs to maximize their therapeutic benefits while minimizing potential negative effects is becoming an increasingly important focus of research.

**Table 1:** An overview of the adverse effects of extracellular vesicles in myocardial infarction.

Cell source	Experimental model	Adverse factors	Key molecule	Related molecular mechanisms	References
Exosomes Derived from Cardiomyocytes	MI Mouse Model, H9C2 Cardiomyocyte Culture	Promotes programmed cell death, exacerbates myocardial injury	miR-328-3p	miR-328-3p induces cell apoptosis by activating the Caspase signaling pathway.	(32)
Exosomes Derived from Cardiomyocytes	MI Mouse Model, Endothelial Cell Culture	Promotes endothelial cell death and inhibits angiogenesis, exacerbates myocardial injury post-myocardial infarction	miR-19a-3p	miR-19a-3p inhibits endothelial cell proliferation and angiogenesis by regulating the expression of HIF-1α.	(33)
Exosomes Derived from Hypoxic Cardiomyocytes	MI Rat Model, Co-culture of Cardiac Fibroblasts with Exosomes from Hypoxic Cardiomyocytes	Promotes cardiac fibroblast apoptosis and inhibits cell proliferation	lncRNA AK139128	Exosomes carrying AK139128 exacerbate cardiac remodeling by affecting the proliferation and apoptosis of cardiac fibroblasts.	(34)
Inflammatory Circulatory Extracellular Vesicles	LAD Rat Model, Pro-inflammatory EV-induced Cardiomyocyte Culture	Promotes cardiomyocyte death	nSMase, NF-kB, TLR4, IL-1α, IL-1β	EVs activate the TLR4-NF-κB axis, leading to cell death.	(35)
Exosomes Derived from Ferroptotic Cardiomyocytes	MI Mouse Model, RAW 264.7 Macrophage Culture	Promotes programmed cell death, exacerbates inflammatory response	Wnt1, β-catenin, NOS2, IL-10	MI-derived exosomes induce M1 macrophage polarization and promote pathological progression of MI through the Wnt/β-catenin pathway.	(39)
Exosomes Derived from M1 Macrophages	MI Mouse Model, Co-culture of miR-155 Mimic-transfected Cardiomyocytes with M1-Exosomes	Inhibits cardiomyocyte proliferation, inhibits angiogenesis, leads to cardiac dysfunction	miR-155	M1-Exosomes deliver miR-155 to inhibit the IL-6R/JAK/STAT3 signaling pathway, thereby suppressing cardiomyocyte proliferation.	(40)
Exosomes Derived from Bone Marrow-Derived Dendritic Cells (BMDCs)	ApoE-/- Mouse Model, Co-culture of Differentiating Dendritic Cells and HUVECs	Promotes inflammation, increases immune cell activation	TNF-α, NF-kB, VCAM-1, ICAM-1	Exosomes activate endothelial cell inflammation and promote the progression of atherosclerosis via TNF-α-mediated NF-κB signaling.	(42)
Plasma Exosomes from Chronic Periodontitis and Carotid Atherosclerosis Patients	ApoE-/- Mouse Model, Culture of HUVECs and HAECs with miR-155-5p Exosomes	Promotes plaque formation, exacerbates vascular burden	miR-155-5p	Exosomes carrying miR-155-5p enhance vascular permeability and angiogenesis, promoting the development of carotid atherosclerosis.	(45)
Exosomes Derived from Adipose Tissue	ApoE-/- Mouse Model	Promotes release of inflammatory factors	miR-34a, Klf4	miR-34a promotes macrophage polarization to the M1 phenotype by inhibiting Klf4, resulting in systemic inflammation and metabolic dysfunction.	(47)
Endothelial Microparticles (EMPs)	Human Aortic Endothelial Cell (hAEC) Culture	Promotes atherosclerosis and thrombosis	EMP, p38 MAPK	Inhibition of p38 MAPK signaling significantly reduces TNF-α-induced EMP generation.	(50)
Endothelial Microparticles (EMPs)	Human Umbilical Vein Endothelial Cell (HUVEC) Culture	EMPs reduce endothelial cell proliferation and increase apoptosis	EMP, Mn-TBAP	EMPs impair HUVEC angiogenesis on Matrigel substrates.	(52)
Exosomes Derived from Cardiomyocytes	MI Rat Model, Isolation of Cardiac Fibroblasts and Cardiomyocytes from Rats	Promotes cardiac fibrosis	miR-208a, Dyrk2	miR-208a targets Dyrk2, promoting the proliferation and myofibroblast differentiation of cardiac fibroblasts.	(54)
Exosomes Derived from CD4+ T Cells	MI Mouse Model, Cardiomyocyte Culture with Cardiac Fibroblasts	Promotes cardiac fibrosis	miR-142-3p	miR-142-3p promotes cardiac fibrosis via the WNT signaling pathway.	(55)

**Table 2:** An overview of the therapeutic implications of extracellular vesicles in myocardial infarction.

Cell source	Experimental model	Therapeutic effect	Key molecule	Related molecular mechanisms	References
Exosomes Derived from Cardiomyocytes	H9c2 Cardiomyocytes Cultured Under Ischemic Conditions, Primary Cardiomyocytes Co-cultured with Endothelial Cells	Promotes endothelial cell proliferation and sprouting, stimulates capillary structure formation, enhances endothelial cell adhesion complexes and barrier properties, improves angiogenesis post-MI	miR-222, miR-143, MMP	miR-222 and miR-143 in exosomes promote angiogenesis by regulating endothelial cell MMP secretion, while enhancing endothelial cell adhesion and barrier function.	(63)
Exosomes Derived from Endothelial Cells	Mouse Model, Human Microvascular Endothelial Cell Line (HMEC-1) *in vitro* Culture	Promotes endothelial cell migration, angiogenesis, and inhibits the expression of mutated ataxia telangiectasia, promoting vascular formation	miR-214	miR-214 mediates endothelial cell signaling through exosomes, inhibits AT expression, promotes angiogenesis, and prevents aging.	(64)
Exosomes Derived from Endothelial Cells	Rat Model, Rat Aortic Endothelial Cells (RAECs) *in vitro* Culture	Exosome-dependent secretion of HSP70 activates monocytes, causing them to adhere to endothelial cells, thereby regulating endothelial function	HSP70	Ox-LDL and Hcy induce endothelial cells to secrete HSP70, exosome-mediated HSP70 promotes monocyte adhesion, providing a new paracrine mechanism to regulate endothelial function.	(65)
Exosomes Derived from Mesenchymal Stem Cells (MSC)	Mouse MI Model, MSC Exosome Treatment of Cardiomyocytes	MSC exosomes increase miR-21a-5p levels to protect the heart, regulate pro-apoptotic genes (PDCD4, PTEN, Peli1, FasL)	miR-21a-5p	Exosomes deliver miR-21a-5p to cardiomyocytes, regulate gene expression, enhance heart protection effects. Exosomes may also promote angiogenesis, cell proliferation, and cardiac repair.	(66)
Exosomes Derived from Human Umbilical Mesenchymal Stem Cells (hucMSC)	AMI Rat Model, EA.hy926 Cell Culture	Improves heart contractile function, reduces cardiac fibrosis, protects cardiomyocytes from apoptosis, promotes angiogenesis	Bcl-2 family, Ki67	hucMSC-exosomes improve heart contractile function by protecting cardiomyocytes from apoptosis and promoting angiogenesis. Their effects are likely related to regulating Bcl-2 family expression and promoting endothelial cell tube formation and migration.	(67)
Exosomes Derived from Cardiomyocytes	Adult Cardiomyocytes, Hypoxia-Reoxygenation Model	Exosomes release HSP60, enhance immune response and protect cardiomyocytes	HSP60	Exosomes release HSP60 through non-classical secretion pathways, HSP60 binds to TLR4 receptors, activating immune responses and cell protection mechanisms; hypoxic stress enhances HSP60 release through exosomes.	(70)
Exosomes Derived from Cardiac Fibroblasts (CFs)	Co-culture of Neonatal Rat CFs and Cardiomyocytes, Myocardial Infarction Mouse Model	CFs increase cardiomyocyte viability via paracrine signaling, reduce infarct size	TIMP-1	Paracrine protective effects mediated by the PI3K/Akt and ERK1/2 signaling pathways, TIMP-1 protects cardiomyocytes via extracellular vesicle action.	(71)
Exosomes Derived from Cardiac Fibroblasts (CFs)	H9C2 Cardiomyocytes, Mouse IRI Model	CFs exosomes/microparticles protect cardiomyocytes in hypoxia-reoxygenation injury, Postcon amplifies this effect	miR-423-3p	Postcon amplifies the heart protection effect by upregulating miR-423-3p expression in CFs exosomes/microparticles, miR-423-3p targets Rap-2c, regulates H9C2 cell viability and apoptosis.	(72)
Exosomes Derived from Cardiac Progenitor Cells (CPC)	H9C2 Cardiomyocyte Culture	miR-21 exosomes inhibit PDCD4 to protect cardiomyocytes from apoptosis induced by oxidative stress	miR-21, PDCD4	CPC-derived exosomes inhibit cell apoptosis through the miR-21/PDCD4 axis, improving cardiomyocytes’ resistance to oxidative stress.	(73)
Exosomes Derived from MSC	Oxygen-Glucose Deprivation (OGD)-Induced Cardiomyocyte Injury; Mouse MI Model	MSC exosome treatment significantly reduces cardiomyocyte apoptosis, reduces inflammation, enhances heart protection	miR-25-3p	miR-25-3p targets FASL and PTEN to reduce protein levels, inhibit EZH2 and H3K27me3, de-repress eNOS and SOCS3 genes, thereby alleviating MI and providing heart protection.	(75)
Exosomes Derived from MSC	Hypoxic Conditions in Cardiomyocytes; Mouse MI Model	MSC exosomes transfer miR-210, significantly reducing cardiomyocyte apoptosis, improving heart function, reducing infarct size	miR-210	miR-210 targets downstream genes like AIFM3, improving cardiomyocytes’ tolerance to hypoxia and other stresses, thus reducing MI damage.	(76)
Exosomes Derived from Adipose-Derived MSC (adMSC)	Oxygen-Glucose Deprivation (OGD) Treated Mouse Cardiomyocytes, MI Mouse Model	Exosome treatment significantly improves cardiomyocyte viability, reduces apoptosis, fibrosis, and inflammation, improves heart function	miR-671	miR-671 targets TGFBR2 and inhibits Smad2 phosphorylation, alleviating myocardial damage caused by MI, improving cell viability, reducing apoptosis and inflammation.	(77)
CDC-exo	Ang II-Induced Cardiac Hypertrophy and Renal Injury Model	Reduces cardiac hypertrophy, decreases cardiac inflammation and fibrosis, improves kidney function, reduces renal inflammation and fibrosis	EV-YF1	CDC-exo and EV-YF1 improve cardiac and renal function, correlated with changes in IL-10 expression in plasma, heart, spleen, and kidneys, without altering blood pressure. Exosomes and their non-coding RNAs may become new therapies.	(78)
Exosomes Derived from Human Heart-Resident Mesenchymal Progenitor Cells (CPC)	Dox/Trz-Induced Cardiotoxicity Rat Model	Reduces Dox/Trz-induced myocardial fibrosis, inflammatory cell infiltration, protects myocardial function	miR-146a-5p	CPC exosomes protect the heart by inhibiting miR-146a-5p target genes (Traf6, Smad4, Irak1, Nox4, and Mpo), reducing oxidative stress and cell death.	(82)
Exosomes Derived from Bone Marrow MSC (BMMSCs)	MI Rat Heart Failure Model	Improves myocardial injury, reduces cardiomyocyte apoptosis and fibrosis, improves heart failure	miR-30e, LOX1, NF-κB p65/Caspase-9	miR-30e negatively regulates LOX1 expression, inhibits NF-κB p65/Caspase-9 signaling, reducing apoptosis and fibrosis, protecting heart function post-MI, and improving heart failure in rats.	(119)
Exosomes Derived from Bone Marrow MSC (BMSCs)	MI Mouse Model	Improves heart function, reduces cell apoptosis and fibrosis, reduces inflammation	miR-129-5p, HMGB1	miR-129-5p targets HMGB1, suppresses inflammation in MI mouse model, reduces inflammatory cytokines and HMGB1 expression, alleviating apoptosis and fibrosis.	(85)
Exosomes Derived from UC-MSCs	MI-I/R Rat Model	Improves heart function, reduces myocardial fibrosis, promotes angiogenesis and cell proliferation	Exo, Cx43, Ki67, CD31, α-SMA	Exosomes combined with injectable conductive hydrogel improve heart function, promote angiogenesis, and myocardial repair, enhancing cell interaction and proliferation.	(87)
Exosomes Derived from Adipose-Derived MSC (ADSC)	Acute MI (AMI) Rat Model, Hypoxia-Induced H9c2 Cardiomyocyte Model	Reduces damage area in myocardial infarction, reduces myocardial fibrosis and inflammatory cytokines, promotes angiogenesis	miR-126, fibrosis-related proteins, inflammatory cytokines	Exosomes rich in miR-126 reduce inflammation in cardiomyocytes, inhibit fibrosis protein expression, promote angiogenesis and cardiac repair.	(88)
Exosomes from IL-10 Deficient EPCs	MI Mouse Model	Improves heart function, reduces MI scar, enhances angiogenesis, but IL-10 deficient exosomes are less effective	IL-10, ILK, NF-κB	IL-10 deficiency leads to upregulation of ILK protein in exosomes, activating NF-κB pathway and promoting inflammation. Knockdown of ILK in exosomes reduces NF-κB activation and restores myocardial repair.	(94)
Exosomes Derived from hucMSC-exosomes	MI Rat Model, LPS-Stimulated Fibroblast Model	Reduces cardiomyocyte apoptosis, promotes differentiation of fibroblasts to myofibroblasts, alleviates inflammation	α-SMA, TGF-β1, IL-6, TNF-α	Exosomes promote fibroblast differentiation to myofibroblasts, enhance cardiac repair, reduce cardiomyocyte apoptosis and inflammation, reducing inflammatory damage in MI area.	(95)
Exosomes from LPS-Stimulated BMSCs	LPS-Stimulated BMSCs, H9c2 Cardiomyocyte Model	Reduces myocardial inflammation and oxidative stress, inhibits cardiomyocyte apoptosis, increases antioxidant enzyme expression	miR-181a-5p, ATF2	miR-181a-5p targets ATF2, inhibits myocardial inflammation and oxidative stress, reduces cell injury, and promotes cardiac repair.	(96)
Exosomes from Adipose-Derived MSC (adMSC)	OGD Treated Mouse Cardiomyocyte Model, MI Mouse Model	Increases cardiomyocyte viability, reduces apoptosis, fibrosis, and inflammation; improves heart function	miR-671	miR-671 targets TGFBR2, inhibits Smad2 phosphorylation, reducing myocardial damage caused by MI, improving cell viability, reducing apoptosis and inflammation.	(77)

### 3.1 The negative aspect of disease rehabilitation

#### 3.1.1 Induction of programmed cell death

Cardiomyocyte-derived exosomes contain a variety of non-coding RNAs, particularly miRNAs, which regulate apoptosis by targeting different apoptotic genes. As shown in **Figure 2E**, Following myocardial infarction, the secretion of certain paracrine factors in cardiomyocyte-derived exosomes increases, and when these exosomes are taken up by recipient cells, they may exacerbate myocardial injury. Caspases, a family of cysteine proteases, play a crucial role in programmed cell death and inflammation by selectively cleaving specific proteins, thereby inducing apoptosis (31). Research by Huang et al. (32) demonstrated that the levels of miR-328-3p in exosomes secreted by infarcted cardiomyocytes are significantly elevated. This miRNA activates intracellular caspase-related signaling pathways, promoting apoptosis. Infarcted cardiomyocytes can also directly transfer exosomes to adjacent cardiomyocytes, further inducing apoptosis and exacerbating MI. Similar studies have shown that miR-19a-3p is enriched in exosomes derived from infarcted cardiomyocytes. When taken up by endothelial cells, it inhibits endothelial cell proliferation and impairs cardiac function in post-MI mice by targeting the expression of hypoxia-inducible factor-1α (HIF-1α) (33). Notably, hypoxia is a key factor contributing to cardiomyocyte apoptosis following MI. The hypoxic environment also activates transforming growth factor β1 (TGF-β1) and its downstream signaling pathways, regulating the proliferation and apoptosis of cardiac fibroblasts (CFs). Long non-coding RNAs (lncRNAs) also play an important role in exosomes. Hypoxic exposure upregulates the expression of lncRNA AK139128 in both cardiomyocytes and exosomes, which has been found to promote CF apoptosis and inhibit proliferation both *in vitro* and *in vivo*, thereby aggravating myocardial injury after MI (33, 34).

Additionally, circulating inflammatory EVs play a critical role in the acute and chronic phases of MI. One study found that inhibiting neutral sphingomyelinase (nSMase) significantly reduced inflammatory EVs and cytokines, improving left ventricular ejection fraction and enhancing cardiac function post-MI. Furthermore, EVs induce cardiomyocyte death by activating the toll-like receptor 4 (TLR4) -nuclear factor-kappa B (NF-κB) axis, further contributing to myocardial damage (35).

#### 3.1.2 Exacerbation of inflammatory responses and impairment of angiogenesis

After AMI, cardiomyocyte death triggers an inflammatory response, and excessive inflammation leads to extracellular matrix (ECM) degradation and ventricular remodeling (36). From an inflammatory perspective, exosomes secreted by cardiomyocytes during AMI regulate various inflammatory cells. According to **Figure 2F**, AMI induces a transient increase in cardiac EVs, which, upon uptake by monocytes in the ischemic myocardium, modulate and enhance local inflammation (37, 38). Additionally, ferroptosis of cardiomyocytes during AMI reduces miR-106b-3p levels in secreted exosomes, activating the WNT signaling pathway, promoting M1 macrophage polarization, and exacerbating myocardial inflammation (39).

Macrophages play a critical role in the progression of inflammation. In the early phase of AMI, M1 macrophages are recruited to the infarcted myocardium, exhibiting strong phagocytic activity. Multiple factors regulate macrophage phenotype changes post-infarction (40). Liu et al. (41) found that M1-type macrophages release pro-inflammatory M1-derived exosomes (M1-Exos) after MI, which impair angiogenesis, accelerate myocardial damage, and are highly enriched in miR-155. miR-155 is transferred to endothelial cells, downregulating multiple target genes involved in inflammation, inhibiting angiogenesis, and leading to cardiac dysfunction. M1-Exos also suppresses related signaling pathways, reducing the angiogenic capacity of endothelial cells, exacerbating the myocardial injury and impeding recovery (40). Additionally, dendritic cell (DC)-derived exosomes recruit and activate immune cells post-MI, promoting the release of inflammatory factors. Advanced experiments have demonstrated that mature DCs contribute to endothelial inflammation via exosomes. DC-derived exosomes (DC-Exos) from bone marrow-derived DC culture medium stimulate human umbilical vein endothelial cells (HUVECs) and mature DC-Exos regulate the NF-κB pathway, increasing HUVEC inflammation (42). Mast cell-derived exosomes, containing pro-inflammatory factors, activate lymphocytes and may contribute to inflammation initiation and amplification. Mast cells can also promote atherosclerotic plaque rupture, leading to AMI (43, 44).

Moreover, endothelial cell-derived exosomes (EC-Exos), depending on their origin and miRNA composition, can have both protective and detrimental effects on the cardiovascular system. While they offer protective effects against vascular injury, they may also contribute to plaque formation, increasing vascular burden. Under oxidized low-density lipoprotein (ox-LDL) stimulation, HUVECs secrete miR-155-enriched exosomes, which promote the transition of monocytes/macrophages from the anti-inflammatory M2 phenotype to the pro-inflammatory M1 phenotype, exacerbating atherosclerotic plaque formation (45).

Adipose-derived exosomes (Ad-Exos) are taken up by macrophages in adipose tissue. Triglycerides within Ad-Exos are hydrolyzed into fatty acids by macrophages and released to maintain systemic metabolic homeostasis. However, under conditions of excessive fat accumulation, this balance is disrupted, leading to macrophage activation, increased inflammatory cytokine release, and systemic insulin resistance. Studies have shown that exosomes isolated from visceral adipose tissue of high-fat diet-fed ApoE-/- mice downregulate ATP-binding cassette transporters (ABCA1 and ABCG1), impairing cholesterol efflux and significantly promoting M1 macrophage foam cell formation and pro-inflammatory factor (TNF-α and IL-6) expression, thereby exacerbating atherosclerosis (46). Another study identified miR-34a as a key regulatory miRNA in Ad-Exos, which transmits nutritional overload signals to resident adipose macrophages. By inhibiting the expression of the transcription factor Krüppel-like factor 4 (Klf4), miR-34a promotes macrophage polarization towards the inflammatory M1 phenotype, aggravating obesity-induced systemic inflammation and metabolic disorders (46, 47).

Beyond exosomes, microparticles also have pro-inflammatory effects and contribute to endothelial dysfunction, promoting atherosclerosis and thrombosis, which are closely associated with AMI progression (48). Endothelial microparticles (EMPs) express adhesion molecules on their surface, facilitating leukocyte aggregation and enhancing their transmigration across endothelial junctions. EMPs activate NF-κB, upregulating Intercellular Adhesion Molecule 1 (ICAM-1) expression, a process that can be inhibited by NF-κB antagonists, suggesting a role in ICAM-1 upregulation via the NF-κB pathway (49). Microparticle release is linked to IL-6 production, with EMPs promoting inflammatory cytokine release in a positive feedback loop. The p38 mitogen-activated protein kinase (p38 MAPK) pathway is critical in producing pro-inflammatory EMPs (50). Furthermore, EMPs inhibit nitric oxide production, impair endothelial relaxation, and increase oxidative stress in a dose-dependent manner (51). Mezentsev et al. (52) found that prolonged exposure to and higher concentrations of EMPs reduce endothelial cell proliferation, increase apoptosis, and impair repair capacity, ultimately leading to endothelial dysfunction. Additionally, leukocyte-derived microparticles (LMPs) participate in all stages of atherosclerosis, promoting inflammation and thrombosis, further contributing to AMI progression.

#### 3.1.3 Enhancement of cardiac fibrosis

Cardiac fibrosis, primarily mediated by activated CFs, contributes to adverse cardiac remodeling and results from various forms of cardiac injury (53). As illustrated in **Figure 2G**, Cardiomyocyte-derived exosomes can influence cardiac fibrosis. Hypoxic cardiomyocytes secrete exosomes enriched with miR-208a into fibrotic cardiac tissue, where CF proliferation and differentiation are promoted into myofibroblasts, exacerbating cardiac fibrosis and further impairing cardiac function (54).

Dendritic cells, as key antigen-presenting cells, also play a role in fibrosis. Cai et al. discovered that CD4+ T cells release exosomes enriched with miR-142-3p, which aggravates cardiac fibrosis and leads to post-MI cardiac dysfunction (55). miR-142-3p directly targets and inhibits the WNT signaling pathway regulator APC, thereby activating the WNT pathway and stimulating CF activation. During cardiac injury, activated macrophages regulate fibroblast differentiation into myofibroblasts through miR-155-enriched exosomes, further driving fibrosis progression (56).

Fibroblast-derived exosomes are also implicated in cardiac fibrosis. These exosomes carry bioactive molecules, including miRNAs and proteins, that influence cardiomyocytes, endothelial cells, and immune cells, thereby accelerating fibrosis. They promote fibrosis by regulating CF proliferation, migration, and ECM protein synthesis and deposition (57). Additionally, fibroblast-derived exosomes may interact with cardiomyocytes, modulating their function, promoting apoptosis, or triggering cellular transformation, thereby worsening myocardial fibrosis (54). Endothelial cell-derived exosomes transmit signals related to vascular function, inflammation, or injury repair, influencing CF migration and proliferation (58). Macrophage-derived exosomes regulate local inflammation and tissue repair, further enhancing CF proliferation, migration, and secretion profile changes, thereby stimulating the secretion of fibroblast-derived exosomes (53).

### 3.2 The positive aspect of disease rehabilitation

In addition to the aforementioned detrimental effects, EVs have been shown to alleviate cardiac dysfunction effectively. Exosomes can be secreted by various cell types, including cardiomyocytes, endothelial cells, cardiac fibroblasts, cardiac progenitor cells, and mesenchymal stem cells (59–61). These exosomes play a crucial role in cardioprotection by promoting angiogenesis, inhibiting myocardial fibrosis, reducing cardiomyocyte apoptosis, suppressing inflammatory responses, and improving cardiac function. Furthermore, the miRNAs and proteins contained within exosomes regulate biological signaling pathways, thereby influencing various physiological and pathological processes in the body. Exosomes from different cellular sources have a wide range of biological functions, which offer great promise for their application in the prevention and treatment of AMI (62).

#### 3.2.1 Promotion of angiogenesis

Cardiomyocytes and endothelial cells maintain close communication, as detailed in **Figure 2A**. Ribeiro-Rodrigues et al. (63) were the first to report that ischemic cardiomyocytes secrete exosomes that influence endothelial cell function and promote angiogenesis. One study confirmed that ischemic cardiomyocyte-derived exosomes protect the myocardium from oxidative damage while stimulating endothelial cell proliferation and sprouting, facilitating new blood vessel formation. Further analysis of miR-143 and miR-222 in exosomes revealed that exosomes from ischemic cardiomyocytes promote angiogenesis both *in vitro* and *in vivo*, underscoring the significant role of intercellular signaling in vascular regulation. Van Balkom et al. (64) demonstrated that miR-214 plays a central role in endothelial cell-derived exosome-mediated signaling. Endothelial cells release miR-214-enriched exosomes, which suppress capillary dilation in target cells, regulate cell migration, and enhance angiogenesis. Zhan et al. (65) further confirmed that ox-LDL and homocysteine induce endothelial cells to release exosomes enriched with heat shock protein 70 (HSP70). These endothelial cell-derived exosomes activate monocyte-endothelial adhesion and upregulate HSP70 expression, providing a novel paracrine mechanism for maintaining vascular endothelial integrity and promoting neovascularization.

Mesenchymal stem cell-derived exosomes (MSCs-Exos) play a crucial role in cardioprotection and angiogenesis. Luther et al. (66) identified miR-21a-5p as a cardioprotective miRNA transferred via exosomes from bone marrow-derived mesenchymal stem cells (BM-MSCs) to cardiomyocytes, promoting angiogenesis. Zhao et al. (67) injected human umbilical cord-derived MSC exosomes (hUC-MSC-Exos) into AMI model rats via the tail vein and observed significant improvement in cardiac contractile function, inhibition of myocardial fibrosis, and enhanced cell proliferation and angiogenesis. Similarly, Ma et al. (68) used adenovirus-transfected hUC-MSCs to isolate and inject exosomes into an AMI model, confirming their ability to promote endothelial cell proliferation and significantly improve cardiac function. Adipose-derived mesenchymal stem cell exosomes (ADSC-Exos) also contribute to angiogenesis by modulating miR-155 expression, improving endothelial cell function, promoting blood vessel formation, and protecting ischemic myocardium from ischemia-reperfusion injury. Additionally, platelet-derived microparticles (PMPs), released by activated platelets and enriched with coagulation-related proteins, promote coagulation, hemostasis, and thrombosis (24, 69). These microparticles interact with endothelial cells to facilitate vascular regeneration and repair, potentially playing a vital role in AMI vascular recovery, particularly restoring damaged vascular function and improving myocardial perfusion.

#### 3.2.2 Inhibition of programmed cardiomyocyte death

Under ischemic and hypoxic stress conditions, cardiomyocytes actively secrete exosomes enriched with specific bioactive cargos, including miRNAs, lncRNAs, and stress-responsive proteins. These exosomes not only mediate intercellular transmission of stress signals but also exert cardioprotective effects by regulating apoptosis-related pathways and mitigating myocardial injury. Notably, certain proteins carried by exosomes, such as heat shock proteins and tumor necrosis factor superfamily member 10 (TNFSF10), play critical roles in modulating apoptosis and immune responses, and have increasingly been identified as promising targets in cardioprotection research (60). Gupta et al. (70) were the first to isolate exosomes containing heat shock protein 60 (HSP60) from adult rat cardiomyocytes. They found that under hypoxic conditions, HSP60 binds to the cardiomyocyte outer membrane, forming a protective barrier that sequesters excessive HSP60, thereby reducing cytotoxicity and inhibiting cardiomyocyte apoptosis.

Recently, tissue inhibitors of metalloproteinases-1 (TIMP-1)have emerged as a key regulator in cardiovascular disease research. Studies have explored the protective role of TIMP-1 in cardiac fibroblast-derived exosomes during MI. Abria et al. (71) injected cardiac fibroblast-derived exosomes into a rat MI model and observed a significant reduction in infarct size and cardiomyocyte apoptosis. This protective effect is thought to be mediated by TIMP-1, which exerts paracrine functions to inhibit fibrosis and mitigate myocardial injury. Luo et al. (72) conducted co-culture experiments and found that cardiac fibroblast proliferation significantly increased under hypoxia-reoxygenation conditions, effectively protecting cardiomyocytes from damage. Their study indicated that cardiac fibroblast-derived exosomes play a cardioprotective role during ischemia-reperfusion injury via the miR-423-3p/RAP2C signaling pathway, inhibiting cardiomyocyte apoptosis.

As shown in **Figure 2B**, excessive reactive oxygen species (ROS) in the ischemic region of AMI are a major cause of cardiomyocyte apoptosis and death. Xiao et al. (73) demonstrated that oxidative stress enhances the production of miR-21 in exosomes derived from cardiac progenitor cells. miR-21 inhibits PDCD4 expression, protecting cardiomyocytes from oxidative stress-induced apoptosis, thus providing a new molecular mechanism for cardioprotection. Additionally, Barile et al. (74) discovered that exosomes from cardiac stem cells contain pregnancy-associated plasma protein-A (PAPP-A), which hydrolyzes IGFBP-4 to release insulin-like growth factor 1 (IGF-1). This activates IGF-1R signaling, leading to phosphorylation of intracellular Akt and ERK1/2, inhibition of caspase activation, and reduced cardiomyocyte apoptosis. Their findings suggest that the cardioprotective effects of cardiac stem cell-derived exosomes are associated with PAPP-A-mediated IGF-1 release.

Peng et al. (75) found that in an AMI mouse model, mesenchymal stem cell-derived exosomes overexpressing miR-25-3p downregulate Fas Ligand (FASL) and phosphatase and tensin homolog (PTEN) expression, thereby suppressing cardiomyocyte apoptosis. Other studies have shown that mesenchymal stem cell-derived exosomes reduce infarct size and improve post-AMI cardiac function (76). The underlying mechanism may involve miR-210, which targets AIFM3, pAKT, and p-p53, regulating apoptosis and enhancing hypoxic cardiomyocyte survival. Furthermore, *in vivo* studies on adipose-derived mesenchymal stem cell exosomes revealed that they improve cardiomyocyte viability, reduce apoptosis, and attenuate both myocardial fibrosis and inflammation. This effect is believed to be mediated by exosomal miR-671, which targets TGFBR2, reducing Smad2 phosphorylation and thereby exerting anti-fibrotic and anti-apoptotic effects (77).

#### 3.2.3 Delaying myocardial fibrosis progression

Elevated levels of angiotensin II induce heart failure and exacerbate the progression of cardiovascular diseases. Exosomes derived from cardiomyocytes can inhibit myocardial fibrosis by regulating the expression of inflammation-related factors, as detailed in **Figure 2C**. Cambier et al. (78) investigated the mechanistic role of cardiomyocyte-derived EVs using a long-term angiotensin II (Ang II) infusion-induced cardiac hypertrophy model established in C57BL/6J mice. Their study revealed that these exosomes modulate the expression of the anti-inflammatory cytokine interleukin-10 (IL-10), thereby alleviating myocardial hypertrophy, reducing cardiac inflammation, and mitigating fibrosis. Additionally, miR-208a, found in cardiomyocyte-derived exosomes, is upregulated in MI models and redox enzyme-induced cardiomyopathy in rats, demonstrating its ability to inhibit myocardial fibrosis and improve cardiac function.

Beyond cardiomyocyte-derived exosomes, cardiac stem cells and progenitor cells (CPCs) play significant cardioprotective roles in AMI through multiple pathways. Cardiac homing peptides (CHPs) are a class of small peptides capable of specifically recognizing and binding to injured myocardial tissue, typically identified through *in vivo* phage display techniques. By targeting endothelial or stromal molecules associated with myocardial injury, CHPs enable the precise delivery of therapeutic agents to diseased cardiac regions. They have been widely employed to enhance the cardiac accumulation of exosomes, drugs, or nanocarriers, thereby improving therapeutic efficacy while minimizing off-target effects (79). Studies have shown that exosomes released by cardiac stem cells can bind to CHP, enhancing their targeted therapeutic effects and reducing post-infarction fibrosis and smaller infarct scars (80). CPC-derived exosomes are highly enriched with miR-146a-5p, which inhibits the deposition of collagen type I in the interstitial matrix, preventing anthracycline/trastuzumab-induced myocardial fibrosis and playing a crucial role in myocardial repair and regeneration (81, 82). He et al. (83) found that CPC-derived exosomes promote regulatory T cell (Treg) differentiation in MI mice, reducing myocardial damage, potentially through enhanced mTOR activity. Moreover, cardiosphere-derived cell (CDC)-secreted exosomes (CDCex) are also rich in miR-146a-5p and have been shown to reduce myocardial fibrosis by inhibiting the expression of pro-inflammatory cytokines and transcription factors (84).

Mesenchymal stem cells (MSCs), commonly derived from bone marrow, also exhibit anti-fibrotic effects through exosome secretion. Studies have demonstrated that exosomes derived from bone marrow MSCs (BMSC-Exo) overexpressing miR-30e can ameliorate myocardial infarction in rats by inhibiting LOX-1 expression and downregulating NF-κB p65/Caspase-9 signaling, thereby reducing myocardial pathological damage and fibrosis (85). Similarly, BMSC-Exo overexpressing miR-129-5p exerts cardioprotective and anti-fibrotic effects in MI models. Furthermore, BMSC-Exo stimulated by lipopolysaccharides (LPS) reduces inflammatory factor expression, improves myocardial contractility, and decreases fibrosis in MI mice. Hypoxia-treated BMSC-Exo, with increased miR-210 expression, has been found to attenuate fibrosis (86). Exosomes from umbilical cord-derived MSCs (UMSC-Exo) delivering circHIPK3 have been shown to reduce infarct zone fibrosis in MI mice (87). Adipose-derived MSCs (ADSCs) overexpressing miR-126 decrease fibrosis-related protein expression in H9c2 cells, alleviating cardiac fibrosis in MI rats (88).

Exosomes derived from ADSCs-Exo have demonstrated superior cardioprotective and anti-fibrotic effects compared to exosomes from unspecified or other stem cell sources in multiple studies. At the molecular level, ADSCs-Exo exert their beneficial effects primarily by overexpressing miR-146a, which downregulates EGR1 expression and suppresses the activation of the TLR4/NF-κB signaling pathway (89). This results in a marked reduction in post-infarction inflammation and cardiac fibrosis, thereby achieving better therapeutic outcomes than unmodified exosomes. At the pathological level, ADSCs-Exo significantly reduce the mRNA levels of multiple fibrosis-related markers, such as COL1A1 and α-SMA, in models of cardiotoxicity induced by doxorubicin and trastuzumab, indicating a more potent anti-fibrotic capacity (90, 91). In terms of immunomodulation, ADSCs-Exo promote macrophage polarization toward the M2 phenotype via activation of the S1P/SK1/S1PR1 signaling pathway, thereby contributing to myocardial microenvironmental remodeling, attenuating inflammation, and enhancing their anti-fibrotic and cardioprotective functions (91). Additionally, ADSCs-Exo have been shown to upregulate SIRT1 expression, leading to a reduction in infarct size and atrial fibrosis in AMI models, highlighting their greater potential in promoting tissue repair and functional recovery (92).

#### 3.2.4 Anti-inflammatory effects

In the field of inflammation research, exosomes derived from HUVECs and human coronary artery endothelial cells (HCAECs) have been shown to modulate inflammatory responses and induce monocyte activation and migration (93). As shown in **Figure 2D**, recent studies have revealed that exosomes secreted by endothelial cells from IL-10 knockout mice lack pro-angiogenic and cardiac repair properties. These exosomes exhibit upregulated expression of integrin-linked kinase (ILK), which activates NF-κB-mediated inflammatory genes in recipient cells. Suppression of ILK expression can rescue the loss of repair activity caused by inflammation (94). In the later stages of MI, M2 macrophages play an anti-inflammatory and reparative role in myocardial tissue. Exosomes from hypoxic cardiomyocytes have been shown to polarize macrophages towards the M2 phenotype, thereby alleviating cardiomyocyte injury, although the underlying mechanisms remain unclear and warrant further investigation. Research by Shi et al. (95) demonstrated that exosomes released by human umbilical cord mesenchymal stem cells (HUCMSCs) can suppress post-MI inflammatory responses and protect cardiomyocytes. Injection of these exosomes into an animal model of AMI resulted in increased myofibroblast density in the infarct zone, further alleviating inflammation. Additionally, studies have shown that exosomes derived from BM-MSCs overexpressing miRNA-181a-5p attenuate inflammation and oxidative stress by downregulating ATF2 expression (77, 96). Exosomes derived from adipose-derived mesenchymal stem cells enhance cardiomyocyte viability, reduce apoptosis, and mitigate myocardial fibrosis and inflammation both *in vitro* and *in vivo*. These effects are potentially mediated by targeting TGFBR2 by exosome-carried miR-671, which reduces Smad2 phosphorylation (77).

## 4 Diagnostic and therapeutic potential of extracellular vesicles as multifunctional carriers in acute myocardial infarction

**Figure 3:**
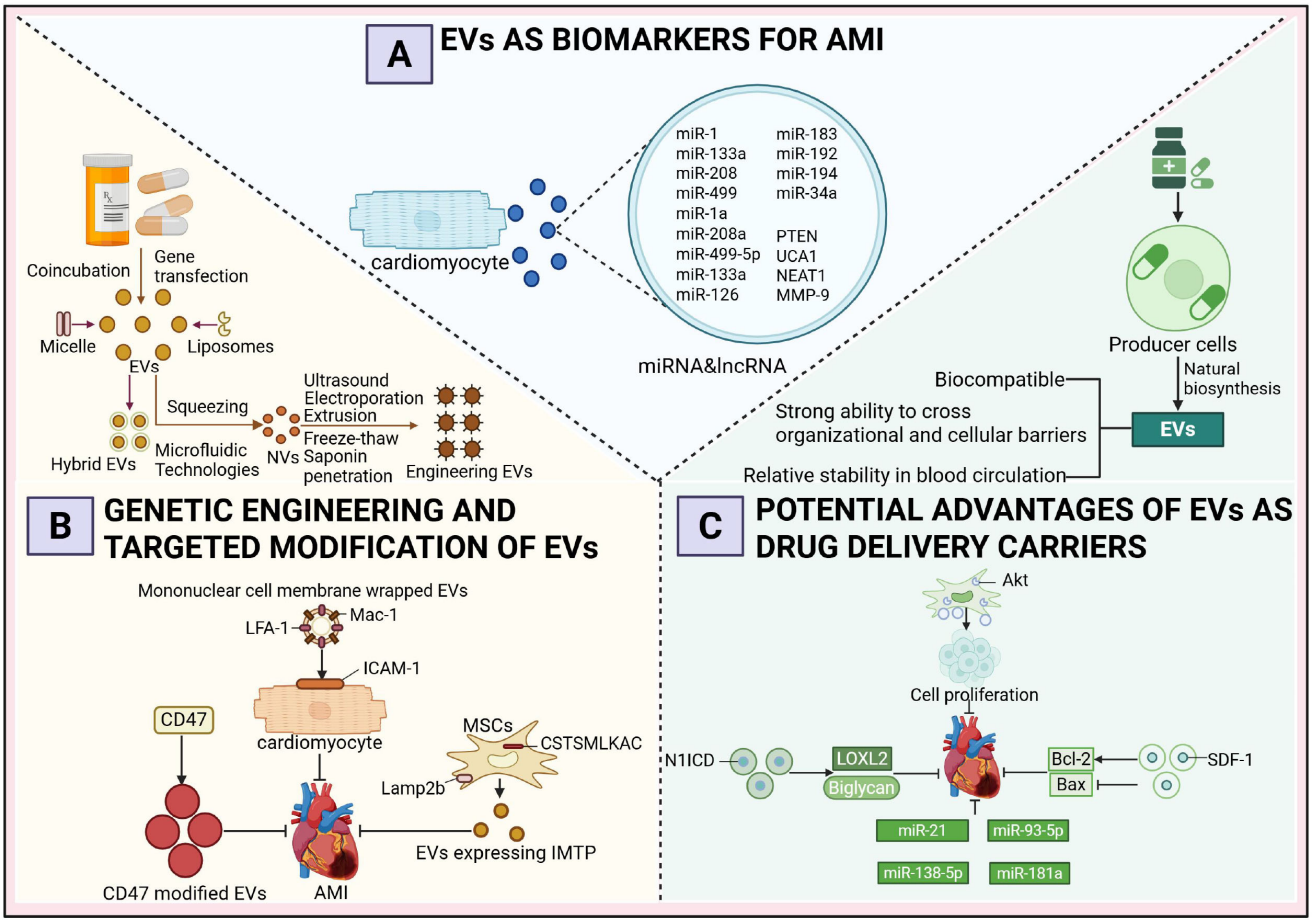
Diagnostic and therapeutic potential of extracellular vesicles as multifunctional carriers in acute myocardial infarction. **(A)** Extracellular Vesicles as Biomarkers of Acute Myocardial Infarction. **(B)** Genetic Engineering and Targeted Modification of Extracellular Vesicles. **(C)** The Prospective Advantages of Extracellular Vesicles as Drug Delivery Vehicles.

EVs have emerged as a promising therapeutic vehicle for AMI, attracting considerable research attention and yielding promising results. EVs have been validated as significant biomarkers for diagnosing and treating AMI. As illustrated in **Figure 3**, EVs have the capacity to transport a diverse array of nucleic acids and proteins into recipient cells, thereby influencing the phenotype and functionality of these cells. This unique characteristic positions EVs as a potentially advantageous drug delivery platform. However, the challenge of achieving precise targeting of EVs to specific recipient cells *in vivo* remains a critical issue that requires further investigation and innovation.

### 4.1 Extracellular vesicles as biomarkers of acute myocardial infarction

Extensive research has demonstrated that AMI patients treated within 12 hours exhibit a 50% reduction in mortality compared to those with delayed intervention, emphasizing the critical importance of early and accurate diagnosis to facilitate timely treatment, minimize myocardial damage, and prevent complications such as heart failure or sudden cardiac death (97, 98). Although cardiac troponin (cTn) is regarded as the gold standard for AMI diagnosis, its elevation in acute non-ACS conditions and chronic diseases highlights the need for more specific and sensitive biomarkers for early AMI detection (99, 100). In recent years, miRNAs and lncRNAs encapsulated within exosomes have emerged as promising biomarkers for the early diagnosis of AMI. Several miRNAs are presumed to originate predominantly from cardiomyocytes, a hypothesis primarily supported by data obtained from animal models. In AMI animal models, particularly in mice subjected to coronary artery ligation, researchers have employed cardiomyocyte-specific promoter-driven reporter systems or exosome-tracking techniques to successfully trace the myocardial origin of specific miRNAs (101). For instance, miR-1a, miR-208a, and miR-499-5p are markedly elevated in the circulation of mice following AMI and are known to be highly enriched in myocardial tissue under physiological conditions (102). Moreover, these miRNAs are rapidly released into the plasma within 2 to 4 hours after myocardial injury, with expression dynamics closely mirroring the progression of myocardial damage, further substantiating their cardiomyocyte-derived origin. Notably, miR-133a is predominantly detected in the non-exosomal fraction of plasma, suggesting a vesicle-independent release mechanism (103). In clinical studies, circulating exosomes from AMI patients have been found to contain elevated levels of miR-126, miR-183, and the PTEN gene, while lncRNAs such as UCA1, NEAT1, and MMP-9 are also significantly upregulated. In contrast, miR-21 and miR-204 exhibit downregulated expression patterns. Among these, the expression levels of miR-126 and miR-183 show a positive correlation with the severity of myocardial ischemia, indicating their potential utility in disease assessment. Therefore, exosomal miRNAs and lncRNAs not only hold promise for the early detection of AMI but may also serve as indicators of the extent of myocardial injury. Furthermore, recent findings suggest that in patients who progress to heart failure within one year following AMI, serum levels of miR-192 are significantly elevated, accompanied by a coordinated upregulation of miR-194 and miR-34a within serum exosomes (104). These three miRNAs may serve as potential biomarkers for predicting long-term heart failure risk after AMI.

### 4.2 Genetic engineering and targeted modification of extracellular vesicles

In addition to their role as biomarkers, EVs have been extensively explored for therapeutic applications in AMI through genetic engineering and targeted modification techniques. A growing body of research demonstrates that engineered EVs exhibit enhanced drug-loading efficiency, targeting precision, and tissue retention. Specifically, donor cells are often engineered via co-incubation or gene transfection to incorporate therapeutic agents, while techniques such as extrusion and microfluidics enable the fabrication of EV-like nanovesicles (NVs). Common methods for engineering EVs include ultrasonication, electroporation, freeze-thaw cycles, extrusion, and saponin permeabilization, all of which facilitate the encapsulation of therapeutic cargo into EVs. Furthermore, the anchoring of targeting ligands, peptides, or aptamers onto the EV membrane enhances their homing capabilities, while the fusion of EVs with lipid-based nanoparticles such as liposomes or micelles results in hybrid EVs with tailored properties (105).

In the context of AMI treatment, one study employed a fusion-extrusion technique to coat EVs with monocyte membranes. Following intravenous injection, these membrane-coated EVs exhibited increased interaction with ischemic cardiomyocytes, driven by the upregulation of ICAM-1 on the cardiomyocyte membrane and the enrichment of Mac-1 and LFA-1 on the EV surface. This interaction promoted EV homing to hypoxic myocardium, thereby improving therapeutic efficacy (106). Another study overexpressed IMTP and Lamp2b in mesenchymal stem cells, resulting in the display of IMTP on the membrane of secreted EVs. Intravenous administration of these IMTP-modified EVs in a murine myocardial infarction model led to enhanced accumulation in the infarcted region, prolonged cardiac retention, and superior therapeutic outcomes compared to unmodified EVs (107). Additionally, CD47-modified EVs, which bind to signal regulatory protein α (SIRPα) to inhibit monocyte-macrophage phagocytosis, demonstrated extended cardiac retention and improved functional recovery in treated mice (108). While most studies focus on genetic engineering to modify EV surfaces, using techniques such as lentiviral transfection raises concerns regarding operational complexity, potential alterations in EV bioactivity, and safety issues such as tumorigenicity (109, 110). To address these limitations, alternative physical or chemical methods have been developed to achieve more precise surface modifications. For instance, chemical conjugation of tissue-specific antibodies or homing peptides onto the EV membrane *in vitro* has been shown to significantly enhance targeting efficiency, offering a safer and more controllable approach to EV engineering. These advancements underscore the transformative potential of engineered EVs in AMI therapy while addressing critical challenges in their development and application (110).

### 4.3 The prospective advantages of extracellular vesicles as drug delivery vehicles

EVs have emerged as promising nanocarriers for treating AMI due to their ability to transport a diverse array of therapeutic molecules, including proteins and miRNAs, which collectively enhance myocardial repair, promote angiogenesis, reduce apoptosis, and inhibit fibrosis. The intrinsic properties of EVs, such as excellent biocompatibility, robust tissue and cellular barrier penetration, and relative stability in the systemic circulation, have driven extensive research into their potential as drug delivery vehicles. Currently, two primary strategies are employed for drug loading into EVs: (1) integrating therapeutic agents into producer cells, utilizing their natural biogenesis pathways to yield drug-loaded EVs, and (2) isolating EVs from various sources (e.g., cultured cells, human blood, or milk) and subsequently incorporating therapeutic molecules using biotechnological methods (111, 112).

Studies have demonstrated the therapeutic efficacy of engineered EVs in AMI models. For instance, Xuan et al. (113) engineered MSCs to overexpress N1ICD, generating N1ICD-enriched EVs that, when injected into the peri-infarct zone of AMI mice, significantly reduced infarct size and fibrosis while improving cardiac function. This effect was attributed to N1ICD-mediated upregulation of LOXL2 and Biglycan, which promoted angiogenesis and attenuated cardiomyocyte apoptosis. Similarly, Ma et al. (68) enhanced the therapeutic potential of MSC-derived EVs by overexpressing Akt, increasing the enrichment of platelet-derived growth factor D (PDGF-D). These EVs facilitated endothelial cell proliferation and migration, angiogenesis in the peri-infarct region, and cardiomyocyte survival, ultimately improving myocardial regeneration and cardiac function. Another study reported that EVs derived from SDF-1-overexpressing MSCs outperformed unmodified EVs in treating myocardial infarction, as they upregulated Bcl-2, downregulated Bax, and inhibited cardiomyocyte apoptosis while promoting microvascular regeneration in the peri-infarct zone (114). The therapeutic utility of EVs has been further expanded by incorporating nucleic acids. Mao et al. (115) loaded MSC-derived EVs with KLF3-AS1, which sequestered miR-138-5p to alleviate its suppression of Sirt1, thereby reducing hypoxia-induced cardiomyocyte apoptosis and enhancing therapeutic outcomes compared to unmodified EVs. Liu et al. (116) engineered adipose-derived MSC EVs to overexpress miR-93-5p, inhibiting Atg7 and TLR4 expression, and attenuating hypoxia-induced autophagy and inflammation. EVs loaded with miR-93-5p demonstrated superior therapeutic effects compared to unmodified EVs. Wei et al. (117) utilized MSC-derived EVs carrying miR-181a to treat AMI mice, resulting in reduced infarct size, improved cardiac function, and decreased inflammatory cell infiltration. Additionally, Song et al. (118) identified miR-21 as a critical cargo in MSC-derived EVs, which targeted the PDCD4/AP-1 pathway to inhibit apoptosis and activated the PTEN/Akt signaling pathway to stimulate VEGF expression, thereby promoting post-AMI functional recovery.

## 5 Discussion

### 5.1 Dual roles and research highlights of EVs in AMI

(1) EVs exhibit a dualistic role in AMI, capable of exacerbating disease progression by promoting apoptosis, amplifying inflammation, and reducing angiogenesis, while also potentially alleviating cardiac injury. Exosomes derived from various cell types have demonstrated cardioprotective effects, including promoting angiogenesis, inhibiting cardiomyocyte apoptosis, repairing damaged myocardium, and suppressing fibrosis. Furthermore, intercellular communication mediated by EVs provides the molecular foundation for their diagnostic and therapeutic roles in cardiovascular diseases, as well as their cardioprotective effects. (2) Targeted modulation of the ratio of M1 to M2 macrophages in cardiac tissue through small exosomes may serve as a potential strategy for treating myocardial infarction. M1 macrophages, classically activated, and M2 macrophages, alternatively activated, exhibit pro-inflammatory and anti-inflammatory phenotypes, respectively, with their balance being critical for tissue inflammation, injury, and repair. Tissue cells and macrophages interact via EVs, with damaged tissue cells releasing exosomes that promote macrophage activation and polarization. Polarized macrophages, in turn, release exosomes and other factors that exacerbate cellular stress, tissue inflammation, and injury. (3) The role of EVs varies significantly across different phases of myocardial infarction, with the acute phase primarily characterized by repair and inflammation, while the chronic phase is more associated with fibrosis and tissue remodeling. Following AMI, cardiomyocytes, endothelial cells, and macrophages rapidly release EVs that carry bioactive molecules involved in inflammation, apoptosis, and repair. In the acute phase, EVs often carry pro-inflammatory factors such as miR-155 and miR-142-3p, which activate immune responses to promote inflammation and local repair, but they may also mediate cardiomyocyte apoptosis or exacerbate cardiac dysfunction. After the acute phase, the function of EVs shifts more towards cardiac remodeling and fibrosis, driving fibrosis by promoting fibroblast-related activities. In the chronic phase, EVs may carry immunomodulatory molecules such as miR-210 and miR-122 to regulate long-term immune responses. However, during chronic myocardial infarction, EVs may also further deteriorate cardiac structure and function due to persistent inflammation and fibrosis. (4) From a therapeutic perspective, the efficacy of stem cell therapy in cardiovascular diseases has been well-established, and exosome-based cell-free therapies are emerging as a new focus for treating conditions such as myocardial infarction and heart failure. With low toxicity, low immunogenicity and excellent biocompatibility, exosomes are a promising natural drug delivery carrier and are expected to become a new generation of nanoscale drug carriers.

### 5.2 Limitations and Prospects of EVs Involvement in the Progression of AMI

Despite the broad therapeutic prospects of EVs, several limitations are faced in current preclinical research: (1) The processes of exosome extraction and purification are complex, and targeted delivery of biologically active factors remains unresolved. Current isolation methods, such as ultracentrifugation, immunocapture, and density gradient centrifugation, exhibit limitations in efficiency, cost, and scalability. (2) There is no standardized method for drug loading into EVs. Although studies have successfully loaded small molecules such as antibiotics and anti-inflammatory drugs into EVs, current techniques, including electroporation, sonication, and incubation, require improved loading efficiency, stability, and targeting precision. (3) To date, no clinical trials are underway to investigate the use of exosomes for treating MI patients. Research on the long-term safety of EVs remains limited, particularly regarding their immunomodulatory effects across different disease states, which are not yet fully understood.To address these challenges, the roles of EVs in AMI should be further explored through the following strategies: (1) Development of EV isolation and purification technologies that comply with good manufacturing practice standards to ensure consistency and controllability in clinical applications. Establishment of quality control systems, including assessments of purity, composition, and bioactivity, to enhance safety and therapeutic predictability. (2) Investigation of the *in vivo* distribution and persistence of EVs to optimize dosing strategies. Exploration of mild yet efficient drug-loading techniques, such as bioengineering EV membrane proteins to enhance interactions with target cells, combined with nanotechnology, such as modifying specific ligands or antibodies to improve targeted delivery capabilities. (3) In-depth evaluation of the metabolic pathways, potential immune side effects, and long-term safety of different EVs *in vivo*. Simultaneously, large-scale animal experiments and clinical trials should be conducted across diverse disease models to validate their efficacy and identify optimal treatment windows.

## 6 Conclusion

EVs have emerged as pivotal tools for delivering essential biological molecules, demonstrating significant potential in the context of AMI. This review systematically summarizes the mechanisms through which EVs influence AMI, with a particular focus on their dual roles in both disease progression and therapeutic intervention. While EVs can exacerbate pathological processes such as programmed cell death and inflammation, they also hold considerable therapeutic potential by promoting angiogenesis and inhibiting cardiomyocyte apoptosis. By comprehensively examining the biogenesis, release, and uptake mechanisms of EVs, as well as their applications in AMI, this review provides a solid foundation for utilizing EVs as biomarkers, drug delivery vehicles, and therapeutic targets. These insights are poised to advance the clinical translation of EVs in the diagnosis and treatment of myocardial infarction and other cardiovascular diseases, thus contributing to the development of precision medicine.
